# Blimp-1 Contributes to the Development and Function of Regulatory B Cells

**DOI:** 10.3389/fimmu.2019.01909

**Published:** 2019-08-14

**Authors:** Ying-Hsiu Wang, Dong-Yan Tsai, Yi-An Ko, Tsan-Tzu Yang, I-Ying Lin, Kuo-Hsuan Hung, Kuo-I Lin

**Affiliations:** ^1^National Defense Medical Center, Graduate Institute of Life Sciences, Taipei, Taiwan; ^2^Genomics Research Center, Academia Sinica, Taipei, Taiwan; ^3^Graduate Institute of Immunology, College of Medicine, National Taiwan University, Taipei, Taiwan

**Keywords:** regulatory B cells, B10 cells, Blimp-1, IL-10, *Candida albicans*

## Abstract

Regulatory B cells (Bregs) are a B cell subset that plays a suppressive role in immune responses. The CD19^+^CD1d^hi^CD5^+^ Bregs that can execute regulatory functions via secreting IL-10 are defined as B10 cells. Bregs suppress autoimmune and inflammatory diseases, whereas they exacerbate infectious diseases caused by bacteria, viruses, or parasites. Notably, the molecular mechanisms regulating the development and functions of Bregs are still largely unknown. Furthermore, the biological impact of Bregs in fungal infection has not yet been demonstrated. Here, we compared the gene expression profiles of IL-10-producing and –non-producing mouse splenic B cells stimulated with lipopolysaccharide (LPS) or anti-CD40 antibody. Blimp-1, a transcription factor known to be critical for plasma cell differentiation, was found to be enriched in the IL-10-producing B cells. The frequency of Blimp-1^+^ B10 cells was increased in LPS-treated mice and in isolated B10 cells that were stimulated with LPS. Surprisingly, B cell-specific Blimp-1 knockout (Cko) mice, generated by CD19 promoter driven Cre recombinase-dependent deletion of *Prdm1* (gene encoding Blimp-1), showed higher frequencies of B10 cells both in the steady state and following injection with LPS, as compared with control littermates. However, B10 cells lacking Blimp-1 failed to efficiently suppress the proliferation of naïve CD4^+^ T cells primed with anti-CD3 and anti-CD28 antibodies. B10 cells can be stimulated for further differentiation into plasmablasts, and a subset of plasmablasts express IL-10. We found that B10 cells from Cko mice failed to generate both IL-10-non-producing and IL-10-producing plasmablasts. Mechanistically, we found that Blimp-1 can directly suppress *Il-10*, whereas, in the presence of activated STAT3, Blimp-1 works together with activated STAT3 to upregulate *Il-10*. Moreover, we also found that B10 cells improve the clearance of *Candida albicans* infection but worsen the infection mortality. Notably, a lack of Blimp-1 in B10 cells did not change these effects of adoptively transferred B10 cells on fungal infections. Together, our data show that Blimp-1 regulates the generation, differentiation, and IL-10 production of Bregs.

## Introduction

Regulatory B cells (Bregs) negatively regulate immune responses by producing interleukin (IL)-10, IL-35, or transforming growth factor-β (TGF-β) (1–3). Bregs are functionally defined B cell subsets, several Breg populations resulting from different inflammatory environments have been described thus far (4–6). Bregs in murine colitis models express CD1d and produce IL-10 to directly suppress intestinal inflammation (7). In the collagen-induced arthritis (CIA) mouse model, the major IL-10-producing B cells are splenic transitional 2 marginal zone precursor (T2-MZP) B cells, which are CD19^+^CD1d^hi^CD21^hi^CD23^hi^CD24^hi^. The adoptive transfer of these cells isolated from mice that have recovered from arthritis can prevent the induction of arthritis (4). Additionally, an IL-10-producing Breg subset was found in mice with contact hypersensitivity (CHS) and in mice with experimental autoimmune encephalomyelitis (EAE); this subset of CD19^+^CD1d^hi^CD5^+^ cells (termed B10 cells), represents only 3–5% of splenic B cells (5).

Microenvironment plays an important role in directing the development or activation of Bregs. It has been demonstrated that antigen and B cell receptor (BCR) signaling is critical for the early development of Bregs. In the presence of CD40 ligand or Toll-like receptor (TLR) ligands, such as lipopolysaccharide (LPS) or CpG, the development of Bregs can be further optimized (2, 3, 8, 9). IL-21 produced by antigen-specific T cells has a role in the development of IL-10-producing Bregs (9). Furthermore, IL-33 and IL-35 can also induce the generation of Bregs that produce IL-10 and/or IL-35 through the activation of signal transducer and activator of transcription (STAT)1 and STAT3 (10, 11).

In addition to its negative regulation of inflammation and autoimmunity, IL-10 production from Bregs also promotes susceptibility in the early stages of infections with viruses, bacteria, helminths, or parasitic protozoa (12–15). For example, in *Listeria* infection, B10 cells promote bacterial persistence and dissemination. A lack of B10 cells enhances *Listeria* clearance as well as CD4^+^ T cell expansion, which is linked with an increased production of interferon gamma (IFN-γ) and tumor necrosis factor alpha (TNF-α) in macrophages (15). However, the role of Bregs in the clearance of fungal infection has not been demonstrated.

B lymphocyte-induced maturation protein-1 (Blimp-1), encoded by *Prdm1*, is a DNA-binding protein that was first discovered as a transcriptional repressor critical for the differentiation of plasma cells (16, 17). It inhibits the expression of genes involved in B cell commitment or identity, such as B-cell lymphoma 6 (*Bcl6*) and paired box 5 (*Pax5*) (18–20). Blimp-1 is not only required for plasma cell formation and immunoglobulin secretion, it is also important for the maintenance of long-lived plasma cells (17, 21). In addition to regulating B cells, Blimp-1 has also been reported to regulate the homeostasis and function of regulatory T cells (Tregs). Blimp-1-deficient Tregs cannot suppress the formation of colitis in mice and fail to inhibit CD4^+^ T cell proliferation, likely owing to their reduced production of IL-10. Furthermore, *Irf4* was found to act together with Blimp-1 to bind *Il-10* and positively regulate the expression of IL-10 in Tregs (22, 23). Whether Blimp-1 is involved in Breg generation and function is still not known. In this report, we assessed the roles of Blimp-1 in Bregs and revealed that Blimp-1 contributes to the generation and function of Bregs.

## Materials and Methods

### Mice

*Prdm1*^*f*/*f*^CD19^Cre/+^ (Cko) mice and *Prdm1*^*f*/*f*^CD19^+/+^ (Ctrl) mice were described previously (17). R26CreER mice (24) and *Prdm1*-EYFP reporter mice (25) were purchased from The Jackson Laboratory. IL-10-IRES-EGFP knock-in (26) (IL-10 reporter, *tiger*, purchased from The Jackson Laboratory) mice were crossed with Cko or Ctrl mice to generate Cko × *tiger* or Ctrl × *tiger* mice. IL-10-deficient (*Il-10* KO) mice (27), purchased from The Jackson Laboratory, were also crossed with Cko or Ctrl mice to generate Cko × *Il-10* KO or Ctrl × *Il-10* KO mice. C57BL/6 mice were purchased from National Laboratory Animal Center, Taiwan. All mice were housed in a specific pathogen-free facility in the Institute of Cellular and Organismic Biology at Academia Sinica and handled in accordance with the guidelines of the Institutional Animal Care and Use Committee. In some experiments, mice were treated with LPS (1.25 μg/g of body weight, clone O111:B4, Sigma-Aldrich) in 200 μl of sterile phosphate-buffered saline (PBS) by intraperitoneal (i.p.) injection. In some experiments, mice were i.p. injected with 5 μg/g body weight of 4-hydroxytamoxifen (4-OHT, Sigma-Aldrich) for three times, separated by 1-day interval, as previously described (28). The efficiency of inducible deletion of *Prdm1* was examined by genomic PCR using isolated splenic B10 cells. The primer sets for the detection of deleted *Prdm1* allele are: 5′-GAGTGAGAGGCGTTAGG-3′ and 5′-AGTAGTTGAATGGGAGC-3′. *Selp* (P-selectin) fragment amplified by 5′-TTGTAAATCAGAAGGAAGTGG-3′ and 5′-CGAGTTACTCTTGATGTAGATCTCC-3′ was used as internal control.

### Cell Purification and Culture

B cells purified from splenocytes by positive selection using anti-B220 antibody beads (Miltenyi Biotec), were cultured in the complete medium (RPMI 1640 containing 10% FBS, 1% penicillin/streptomycin and 0.1% 2-mercaptoethanol (ME), all from Life Technologies) at the density of 2 × 10^6^ cells/ml. Cells were stimulated with anti-CD40 antibody (2 μg/ml, clone HM40-3, BD Pharmingen) or LPS (10 μg/ml) for 5–48 h. In some experiments, phorbol-12-myristate-13-acetate (PMA, 50 ng/ml, BD Pharmingen), ionomycin (500 ng/ml, Sigma-Aldrich), and monensin (2 μM, eBioscience) were added in culture for the final 5 h before the detection of intracellular IL-10 (29). In microarray analysis, splenic B cells were treated with either LPS or anti-CD40 for 48 h, and IL-10^+^ or IL-10^−^ B cells were then purified by Regulatory B Cell Isolation Kit (Miltenyi Biotec) according to the manufacturer's instructions. To compare the genes differentially expressed in Ctrl and Cko B10 cells, isolated B10 cells from Ctrl and Cko mice were stimulated with anti-CD40. RNA samples were collected at 0 and 48 h after stimulation.

### Microarray and Gene Ontology (GO) Analysis

Total RNA samples extracted from indicated splenic B cells were subjected to Affymetrix GeneChip microarray analysis. In brief, the RNA was amplified, biotin labeled, and purified by using GeneChip 3′ IVT PLUS Reagent Kit (Affymetrix) according to the manufacturer's instructions. Biotinylated cRNA was hybridized to Affymetrix Mouse Genome 430 2.0 Array via Hybridization Oven 645 (Affymetrix), and Affymetrix Fluidics Station 450 (Affymetrix) was used to wash and stain the Chips. The array data were acquired using GeneChip^®^ Scanner 3000 (Affymetrix) and analyzed by GeneSpring GX. In some experiments, total RNA was extracted from Ctrl or Cko B10 cells before and after stimulation with anti-CD40 for 48 h. The microarray results are available under the accession number GSE129260 and GSE133762 in the GEO database. GO analysis of differentially expressed genes was performed using the Biological Networks Gene Ontology (BiNGO) program package with *p* ≤ 0.05 as previously described (30).

### Flow Cytometry

Single-cell suspensions from spleen were stained with the following antibodies: anti-CD19-PEcy7 monoclonal antibody (mAb) (clone 6D5, Biolegend), anti-CD1d-PE mAb (clone 1B1, Biolegend), anti-CD5- Brilliant Violet 421mAbs (clone 53-7.3, Biolegend), anti-CD5-PerCp mAbs (clone 53-7.3, BD Biosciences), anti-CD138-Brilliant Violet 421mAbs (clone 281-2, Biolegend), anti-CD21/35-APC mAb (clone 7E9, Biolegend), anti-CD23-PEcy7 mAb (clone B3B4, Biolegend), anti-B220-APCcy7 mAb (clone RA3-6B2, Biolegend), and anti-CD4-PerCp-Cy5.5 mAbs (clone RM4-5, BD Biosciences), using the protocols described previously (31). For intracellular cytokine staining, after cell surface staining, the cells were fixed and permeabilized by using a Cytofix/Cytoperm kit (BD Biosciences) and stained with anti-IL-10-APC mAb (clone JES5-16E3, BD Biosciences) in accordance with the manufacturer's instructions. The detection of STAT3 phosphorylation was performed as preciously described (32). Briefly, sorted B10 cells were stimulated with LPS (10 μg/ml) for 5–48 h, after which the cells were fixed in Lyse/Fix buffer (containing 7.15% methanol (w/w), 20.35% formaldehyde (w/w), and 15.65% diethylene glycol (w/w); BD Phosflow™) for 10 min at 37°C. After being washed, the cells were permeabilized on ice with Perm Buffer III (BD Phosflow™) for 20 min. The cells were then stained with anti-pSTAT3 (Y705)-Pacific Blue mAb (clone 4/P-STAT3, BD Phosflow™) for 30 min at room temperature. Cells treated with isotype-matched mAbs or splenic B cells from *Il-10* KO mice served as the negative controls. The cells were analyzed using a BD FACSCanto™ II, and, in some experiments, the cells were sorted by a BD FACSAria™ II.

### Co-culture of B10 Cells Together With CD4^+^ T Cells

Naïve CD4^+^ T cells were isolated from murine spleens by using a Naïve CD4^+^ T Cell Isolation Kit (Miltenyi Biotec) and labeled with carboxyfluorescein diacetate succinimidyl ester (CFSE) from a CellTrace™ CFSE Cell Proliferation Kit (Life Technologies). CFSE-labeled naïve CD4^+^ T cells at a concentration of 1~2 × 10^6^ cells/ml were cultured alone or co-cultured with isolated B10 cells (1 × 10^6^ cells/ml) from Ctrl, Cko, Ctrl × *Il-10* KO, or Cko × *Il-10* KO mice with plate-bound anti-CD3 antibody (5 μg/ml, Invitrogen) and soluble anti-CD28 antibody (2 μg/ml, Invitrogen). After 3 days, the cells were collected for an assessment of the CFSE dilution in the CD4^+^ T cells conducted by flow cytometry.

### *Candida albicans* (*C. albicans*) Infection and Fungal Burden Assay

*Candida albicans* (SC5314, a gift from Dr. Betty A. Wu-Hsieh, National Taiwan University) was cultured on yeast extract peptone dextrose (YPD, BD Difco) agar (Bioshop) plates at 30°C. The colonies were harvested and diluted in sterile PBS to a concentration of 2.5 × 10^6^ colony-forming units (CFU)/ml. Mice were infected intravenously (i.v.) with 5 × 10^5^ CFU of *C. albicans* in 200 μl of PBS, and their survival was recorded over time. To assay the fungal burden, the kidneys of *C. albicans*-infected mice were excised and homogenized in 1 ml of PBS with MagNALyser Green Beads (Roche) by MagNALyser (Roche) for 60 s at 6,000 rpm and then cooled for 60 s. The fungal burden was measured by serially diluting the homogenates in 100 μl of PBS, followed by plating them onto YPD agar plates and incubating them at 30°C for 2 days. The numbers of colonies were counted manually. To perform serum transfer experiments, naïve C57BL/6 mice were injected i.v. with 2 × 10^5^ CFU of *C. albicans* in 200 μl of PBS. The blood samples from infected or uninfected mice were collected through submandibular vein 2 weeks after post-infection and pooled. The serum was harvested, and 200 μl of serum from uninfected mice (normal serum) or infected mice (CA serum) were i.v. injected into Ctrl or Cko recipient mice 1 day before *C. albicans* infection.

### Adoptive Transfer of B10 Cells

A total of 1 × 10^6^ isolated (CD19^+^CD1d^hi^CD5^+^) B10 cells from different genotypes of mice were suspended in 200 μl PBS and were then i.v. transferred into recipient mice 1 day before *C. albicans* infection. PBS injection was as a control for cell transfer.

### Enzyme-Linked Immunosorbent Assay (ELISA)

Sorted B10 cells were stimulated with LPS (10 μg/ml). The cell culture supernatants were collected for detection of IL-10 production by using an IL-10 ELISA kit (BD OptEIA) at various timepoints. The reaction was stopped with 2 N H_2_SO_4_ and the absorbance was measured at 450 nm by SpectraMax M2 microplate reader (Molecular Devices).

### RNA Isolation and Reverse Transcription–Quantitative Real-Time PCR (RT-qPCR)

Total RNA was extracted from stimulated B10 cells by RNeasy Plus Mini Kit (QIAGEN) and subjected to generation of cDNA by using High-Capacity cDNA Reverse Transcription Kit (Applied Biosystems) according to the manufacturer's instructions. The quantitative real-time PCR was performed on Applied BiosystemsStepOnePlus™ Real-Time PCR Systems. Primer pairs for *Il-10* with SYBR green system are: 5′-GCTCTTACTGACTGGCATGAGGAT-3′ and 5′-GCTGGTCCTTTGTTTGAAAGAAAG-3′. Primers for detecting *Actin* with SYBR green system are: 5′-GCTGTATTCCCCTCCATCGTG-3′ and 5′-CACGGTTGGCCTTAGGGTTCAG-3′. TaqMan probe for *Prdm1* (Mm 01187285_m1) was purchased from Applied Biosystems. Relative levels of mRNA were normalized to *Actin* expression in each sample.

### Chromatin Immunoprecipitation (ChIP) Assay

To assess the binding of Blimp-1 to the *Il-10* promoter, splenic B cells were stimulated with LPS (2.5 μg/ml) and harvested for a ChIP assay as previously described (20). Briefly, a total of 2 × 10^6^ cells were used along with anti-Blimp-1 antibody (2 μg, Abcam) or an isotype control antibody (IgG, 2 μg, Abcam), and the resulting immunoprecipitated samples were quantified by qPCR using primer sets that specifically amplified a fragment covering Blimp-1 binding sites or within 200 bp of Blimp-1-binding sites in the *Il-10* promoter. The results were normalized using the isotype control groups. The sequences of PCR primers are:

*CIITA*: 5′-GCCACCTTGCAGGGAGAGT-3′ and 5′-AAGCTAAGCAACATGCAAAGAA-3′, *CIITA* 3′UTR: 5′-CCATCATGTCTGGCTAATTTTTCA-3′ and 5′-GGATCACCTGAGGTCAAGAGTTTG-3′, *Il-10* 3′UTR: 5′-CTGCAGTGTGTATTGAGTCTGCT-3′ and 5′-TGGGAACTGAGGTATCAGAGG-3′, *Il-10* −9K: 5′-CTTGAGGAAAAGCCAGCATC-3′ and 5′-TTTGCGTGTTCACCTGTGTT-3′, *Il-10* −0.45K: 5′-GACTTCCGAGTCAGCAAGAAA-3′ and 5′-ACGTGGATAAATGGGCTATTCC-3′, *Il-10* −0.15K: 5′-AGTTCATTCCGACCAGTTCTTT-3′ and 5′-TCCTCCTCCCTCTTCTAAACC-3′.

### Transfection and Luciferase Reporter Assay

The *Il-10* promoter was amplified from the genomic DNA of C57BL/6 mice, and the resulting PCR fragment was cloned into a pGL3 Basic vector (Promega). The detailed cloning procedures and site-directed mutagenesis of the *Il-10* promoter with disrupted Blimp-1- or STAT3-binding sites will be available upon request. B cell transfection was performed essentially as previously described (31). Briefly, 2 × 10^6^ Raji B cells were transfected with various doses of luciferase reporter vectors, vectors expressing Blimp-1 (pCMV-flag-Blimp-1), vectors expressing constitutively activated STAT3 (STAT3-CA) or wildtype STAT3 (kind gift from Dr. Chien-Kuo Lee, National Taiwan University), and 500 ng of pRL-TK as a transfection efficiency control. Transfection was performed by using an Amaxa^®^ Cell Line Nucleofector^®^ Kit V (Lonza) in accordance with the manufacturer's instructions. In some experiments, samples were treated with IL-21 (20 μg/ml, PeproTech). A luciferase assay was performed using a Dual Luciferase Reporter Assay System (Promega) at 48 h post-transfection after harvesting the cells with passive lysis buffer (PLB). Luminescence was measured by a microplate reader (Victor3™, PerkinElmer). The relative fold difference in luciferase reporter activity was calculated by normalization of the ratio of firefly:Renilla luciferase activity to the ratios obtained from control transfection with pGL3B alone and test expression vectors.

### Western Blotting

Western blotting was performed as previously described (33). Briefly, total cell lysates (30 μg) were subjected to SDS-PAGE and western blotting analysis using the following primary antibodies: anti-Blimp-1 (1:1,000 dilution, Abcam), anti-STAT3 (1:500 dilution, Cell Signaling), anti-phospho-STAT3 at Tyr705 (1:500 dilution, Cell Signaling), anti-phospho-STAT3 at Ser727 (1:500 dilution; Cell Signaling), anti-actin (1:2,000 dilution, GenScript), and anti-Flag (1:1,000 dilution, Sigma) antibodies. Secondary antibodies were as previously described (33). Representative blots from at least two independent experiments are shown.

### Statistical Analysis

The statistical significance of each difference was analyzed by using an unpaired Student's *t*-test. Data are shown as the mean ± SEM. The differences in Kaplan-Meier survival curves between two groups of mice were calculated by log-rank (Mantel-Cox) tests (GraphPad Prism 5).

## Results

### The Blimp-1 Expression in Bregs

**Figure 1:**
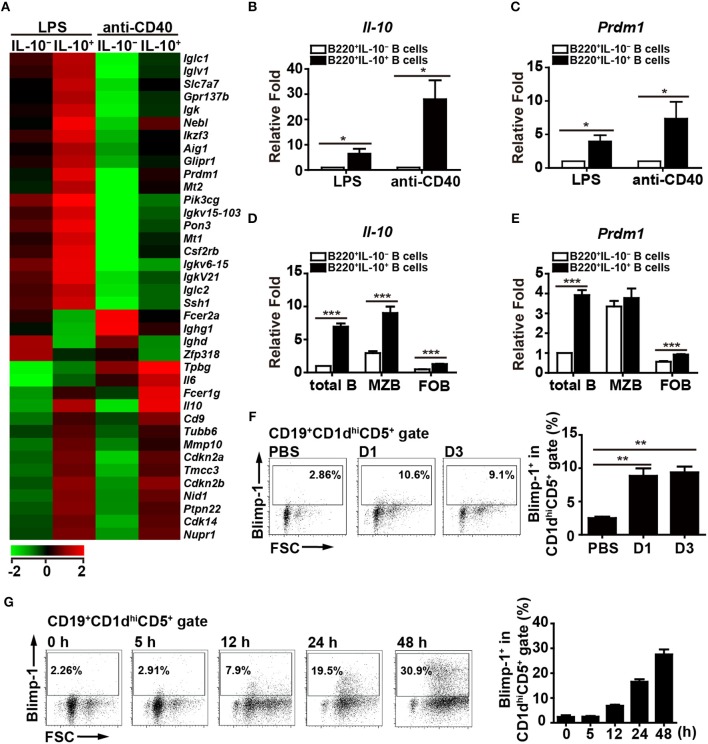
Blimp-1 expression in Bregs. **(A)** IL-10^+^ B cells and IL-10^−^ B cells were purified from C57BL/6 mouse splenocytes and then stimulated with LPS (10 μg/ml) or anti-CD40 antibody (2 μg/ml) for 48 h, after which their mRNA expression profiles were analyzed by microarray. The resulting clustering heat map diagram shows the fold changes in differentially expressed genes with changes of >1.8-fold after stimulation. Data are the mean values from two experiments. **(B,C)** RT-qPCR analysis of *Il-10* **(B)** and *Prdm1* **(C)** mRNA levels in IL-10^+^ B cells and IL-10^−^ B cells isolated from mouse splenocytes. Data are the mean ± SEM (*n* = 3–5 mice per group). **(D,E)** RT-qPCR analysis of *Il-10* **(D)** and *Prdm1* **(E)** mRNA levels in IL-10^+^ cells and IL-10^−^ cells isolated from total B, MZ B and FO B cells treated with LPS. Data are the mean ± SEM (*n* = 5 mice per group). Results are normalized to the IL-10^−^ total B cells. **(F,G)** Blimp-1 expression by CD19^+^CD1d^hi^CD5^+^ B10 cells from *Prdm1*-EYFP reporter mice *in vivo* **(F)** and *in vitro* **(G)**. **(F)** *Prdm1*-EYFP reporter mice were injected with LPS (1.25 μg/g of body weight) and sacrificed at the indicated days post-injection. Splenocytes were isolated at the indicated days and subjected to FACS analysis. Because the results from the PBS injection control group were unchanged at the indicated days, they were pooled in our statistical analysis. **(G)** Splenic CD19^+^ B cells were purified from *Prdm1*-EYFP reporter mice and cultured with LPS (10 μg/ml) for 5–48 h. The frequency of Blimp-1^+^ cells in the CD19^+^CD1d^hi^CD5^+^B10 gate is indicated. The results are from at least two independent experiments and were analyzed using an unpaired Student's *t*-test. Data are the mean ± SEM (*n* = 3–6 mice per group). ^*^p < 0.05, ^**^p < 0.01 and ^***^p < 0.001.

Previous studies showed that stimulation with LPS or anti-CD40 antibody can induce IL-10 production by B cells (29). To investigate which genes are involved in regulating the production of IL*-*10 by B cells, purified splenic B cells were stimulated with LPS or anti-CD40 antibody for 48 h, followed by the isolation of IL-10-producing (IL-10^+^) B cells and IL-10-non-producing (IL-10^−^) B cells for microarray analyses. Thirty-four genes, including *Prdm1*, were found to be upregulated and four genes were found to be downregulated at least 1.8-fold in the IL-10^+^ B cells as compared with the IL-10^−^ B cells in both LPS- and anti-CD40 antibody-stimulated cultures (Supplementary Table 1, Figure 1A). RT-qPCR results confirmed that the *Il-10* mRNA levels were indeed higher in the IL-10^+^ B cells (Figure 1B). Notably, the *Prdm1* mRNA levels were also significantly higher in the IL-10^+^ B cells (Figure 1C). It has been reported that, within splenic B cell subsets, marginal zone (MZ) B cells, but not follicular (FO) B cells, in the inflammatory condition can also differentiate into IL-10-producing cells (34). Sorted MZ (B220^+^CD21^hi^CD23^low^) and FO (B220^+^CD21^low^CD23^hi^) B cells were treated with LPS, followed by isolation of IL-10^+^ cells and IL-10^−^ cells for RT-qPCR analysis. Our results showed that both MZ and FO B cells, particularly MZ B cells, can produce IL-10 after LPS stimulation (Figure 1D), but *Prdm1* mRNA levels appear to be highly enriched in IL-10^+^ total B cells (Figure 1E). These results suggest that, besides MZ and FO B cells, other IL-10-producing B cell subsets, such as Bregs, may have elevated *Prdm1* mRNA levels following LPS stimulation.

Because the role of Blimp-1 in IL-10-producing Breg cells has not been formally reported, we pursued the above finding. To further ensure the expression of Blimp-1 in IL-10^+^ Bregs, *Prdm1-*EYFP reporter mice were intraperitoneally injected with LPS, which can induce the expansion of Breg cells *in vivo* (35). We found that a small portion of Blimp-1^+^ (EYFP^+^) B cells was present in the CD1d^hi^CD5^+^ B10 cells in PBS-injected mice (Figure 1F). More importantly, LPS-injected mice showed an increased frequency of Blimp-1^+^ B10 cells (Figure 1F). Furthermore, splenic B cells from the *Prdm1*-EYFP reporter mice also showed a higher frequency of Blimp-1^+^ cells in the B10 cell gate following LPS stimulation (Figure 1G). Together, our data show that B10 cells express Blimp-1 and that Blimp-1 levels in B10 cells are increased following stimulation, both *in vivo* and *in vitro*.

### IL-10^+^ B Cell Levels Are Increased in *Prdm1*-Deficient Mice

**Figure 2:**
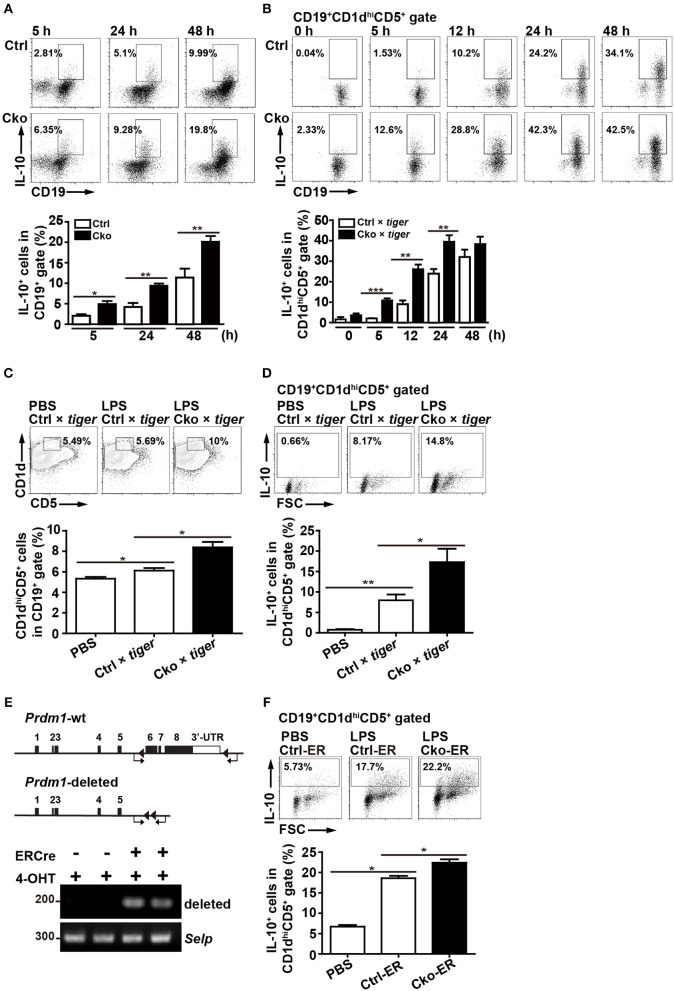
Splenic B10 population in Ctrl and Cko mice. **(A)** Splenic B cells were purified from *Prdm1*^*f*/*f*^CD19^cre/+^mice (Cko) or control littermates (*Prdm1*^*f*/*f*^CD19^+/+^, Ctrl) and then stimulated with LPS (10 μg/ml) for 5–48 h. PMA, ionomycin, and monensin (PIM) were added into culture for the final 5 h for intracellular staining of IL-10. The results represent the frequency of IL-10-producing cells in the CD19^+^ gate. Data are the mean ± SEM (*n* = 3–6 mice per group). **(B)** *Il-10* reporter mice, *tiger*, were crossed with Cko and Ctrl mice. Splenic B cells were purified from the Cko × *tiger* or Ctrl × *tiger* mice and then stimulated with LPS (10 μg/ml) for 5–48 h before use in an analysis of the frequency of GFP^+^ B10 cells. **(C,D)** Cko × *tiger* or Ctrl × *tiger* mice were given LPS (1.25 μg/g of body weight). B10 cells **(C)** and IL-10^+^ (GFP^+^) B10 cells **(D)** in Cko × *tiger* or Ctrl × *tiger* mice were analyzed at the indicated days. A PBS-injected group was used as the control. Data are the mean ± SEM (*n* = 3 mice per group). **(E)** PCR analysis of indicated genomic DNA isolated from splenic B220^+^ B10 cells of Cko-ER and littermate control Ctrl-ER mice 14 days after injection with 4-OHT. Primers used for the detection of *Prdm1* deletion were indicated by arrows. **(F)** Fourteen days after 4-OHT injection, Cko-ER and Ctrl-ER mice were injected with LPS (1.25 μg/g of body weight). IL-10^+^ B10 cells were then analyzed 3 days later by FACS analysis. Data are the mean ± SEM (*n* = 3 mice per group). A PBS-injected group was used as the control. Results are from at least two independent experiments and were analyzed using an unpaired Student's *t*-test. ^*^p < 0.05, ^**^p < 0.01 and ^***^p < 0.001.

Given that one of the most important regulatory functions of B10 cells is to produce IL-10, we next assessed the role of Blimp-1 in IL-10 production in B10 cells. We used *Prdm1*^*f*/*f*^CD19^Cre/+^ (Cko) mice and *Prdm1*^*f*/*f*^CD19^+/+^ (Ctrl) mice to address this question. Purified splenic B cells from Cko and Ctrl mice were stimulated with LPS, then treated with PMA, ionomycin, and monensin (PIM), which facilitate cytoplasmic IL-10 expression (29), for the final 5 h before their use in the detection of cytoplasmic IL-10 by FACS. We found that the frequency of CD19^+^IL-10^+^ B cells was increased after LPS treatment in both the Ctrl and Cko B cell cultures at various timepoints (Figure 2A). Unexpectedly, we found a significantly higher frequency of CD19^+^IL-10^+^ B cells in the Cko culture at all timepoints (Figure 2A).

We next crossed Ctrl and Cko mice with *Il-10*-green fluorescent protein (GFP) reporter mice (called *tiger*) (26) to generate Ctrl × *tiger* and Cko × *tiger* mice. Purified splenic B cells from Ctrl × *tiger* and Cko × *tiger* mice were each stimulated with LPS, after which the frequency of GFP^+^ (IL-10^+^) B cells within gated CD19^+^CD1d^hi^CD5^+^ B10 cells was measured. Similar to the results observed in Figure 2A, the frequency of GFP^+^ (IL-10^+^) B10 cells were higher in cultures of B cells from Cko × *tiger* mice as compared with those from Ctrl × *tiger* mice at various timepoints post-stimulation (Figure 2B). Further, higher frequencies of splenic B10 cells and GFP^+^ (IL-10^+^) B10 cells were found in Cko × *tiger* mice 3 days after an intraperitoneal injection of LPS as compared with LPS-treated Ctrl × *tiger* mice (Figures 2C,D). When Bregs were labeled with 5-bromo-2'-deoxyuridine (BrdU) after 24 or 48 h of LPS stimulation, the frequencies of BrdU^+^ cells in the Cko and Ctrl B cell cultures were comparable (Supplementary Figure 1A), suggesting that the increased number of Bregs may not result from the increased proliferation. The apoptosis rates of Bregs were also determined, but there were no significant differences in these rates between the Ctrl and Cko Bregs (Supplementary Figure 1B). Thus, Blimp-1 negatively regulates the generation of B10 cells as well as the IL-10 production by B10 cells.

To further ensure the role of Blimp-1 in Breg generation, we crossed *Prdm1*^*f*/*f*^ mice with mice carrying the inducible estrogen receptor/cre (ER-cre) in ubiquitous tissues (36, 37). Splenic B10 cells isolated from the inducible *Prdm1* knockout (Cko-ER) mice had deletion of *Prdm1* allele 14 days after injection with 4-hydroxytamoxifen (4-OHT) (Figure 2E). Again, we found that, inducible deletion of *Prdm1* in mice caused increased frequency of IL-10^+^ B10 cells following LPS injection (Figure 2F).

### Blimp-1-Deficient B10 Cells Cannot Inhibit T Cell Proliferation

**Figure 3:**
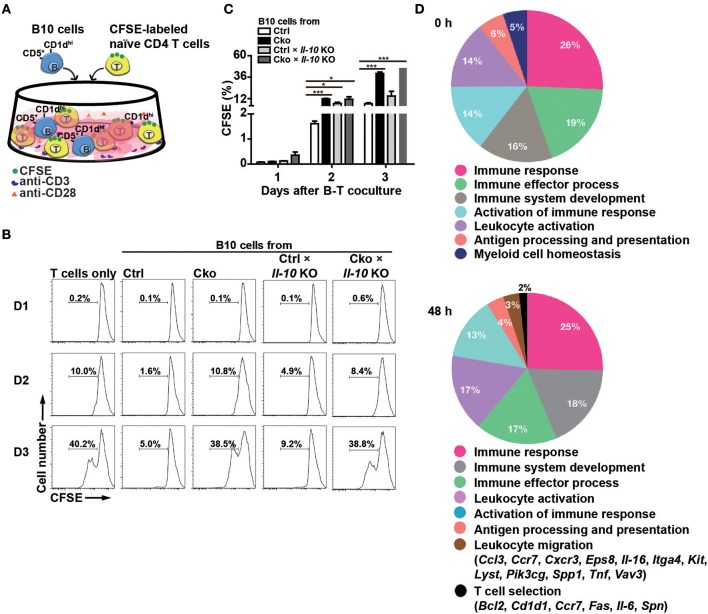
B10 cells lacking Blimp-1 failed to suppress T-cell proliferation. **(A)** Experimental design for the co-culture of B10 cells with anti-CD3-and anti-CD28-stimulated naïve CD4^+^ T cells. CFSE-labeled CD4^+^ T cells in the presence of anti-CD3 and anti-CD28 antibody were co-cultured with B10 cells from mice of the indicated genotypes for 3 days and then analyzed for CFSE dilution by FACS. **(B)** FACS analysis showing the frequency of dividing CFSE-labeled CD4^+^ T cells. Data are representative from three independent biological replicates. **(C)** Statistical results from three experiments in **(B)** are shown. Data were analyzed using an unpaired Student's *t*-test and are the mean ± SEM (*n* = 3 mice per group). ^*^p < 0.05 and ^***^p < 0.001. **(D)** GO analysis showed a significant enrichment for genes in “T cell selection” and “leukocyte migration” in the category of immune system process within the Biological Process ontology in anti-CD40 stimulated Cko B10 cells as compared with genes in anti-CD40 stimulated Ctrl B10 cells. The significant terms in the immune system process within the Biological Process ontology were all listed.

According to previous studies (14, 38–40), Bregs can suppress T cell proliferation. To ascertain whether Blimp-1 is important for the regulatory functions of B10 cells, we crossed Ctrl and Cko mice with *Il-10* KO mice (called Ctrl × *Il-10* KO and Cko × *Il-10* KO, respectively). We performed co-cultures of CFSE-labeled naïve CD4^+^ T cells that had been treated with anti-CD3 and anti-CD28 antibodies together with isolated B10 cells from Ctrl, Cko, Ctrl × *Il-10* KO, or Cko × *Il-10* KO mice (Figure 3A). As expected, B10 cells from the Ctrl mice effectively suppressed the proliferation of anti-CD3-and anti-CD28-stimulated CD4^+^ T cells (Figures 3B,C). However, the proliferation of stimulated CD4^+^ T cells was not significantly affected by co-culture with B10 cells isolated from Ctrl × *Il-10* KO mice (Figures 3B,C). Notably, B10 cells from Cko mice could not efficiently suppress the proliferation of activated T cells (Figures 3B,C). B10 cells from Cko × *Il-10* KO mice still failed to suppress the proliferation of activated T cells (Figures 3B,C). Together, these results suggest that IL-10 produced by B10 cells may not be critical for suppression of T cell proliferation, and that, independent of IL-10 production, Blimp-1 in B10 cells is critical for suppressing T cell proliferation.

To further dissect the possible pathways that conferred the suppressive function of Blimp-1 in Bregs in this context, we performed microarray analysis using anti-CD40 stimulated Ctrl and Cko B10 cells. Two hundred eighty-seven and 649 genes showed at least 1.5-fold changes before and 48 h after anti-CD40 stimulation, respectively, in Cko B10 cells as compared with those in Ctrl B10 cells (Supplementary Table 2). GO analysis of the differentially expressed genes between Ctrl and Cko B cells at each timepoint revealed that genes involved in “leukocyte migration” and “T cell selection” within the GO category of immune system process were significantly enriched in anti-CD40 stimulated Cko B10 cells (Figure 3D).

### B10 Cells Promote Fungal Clearance Independently of Blimp-1

**Figure 4:**
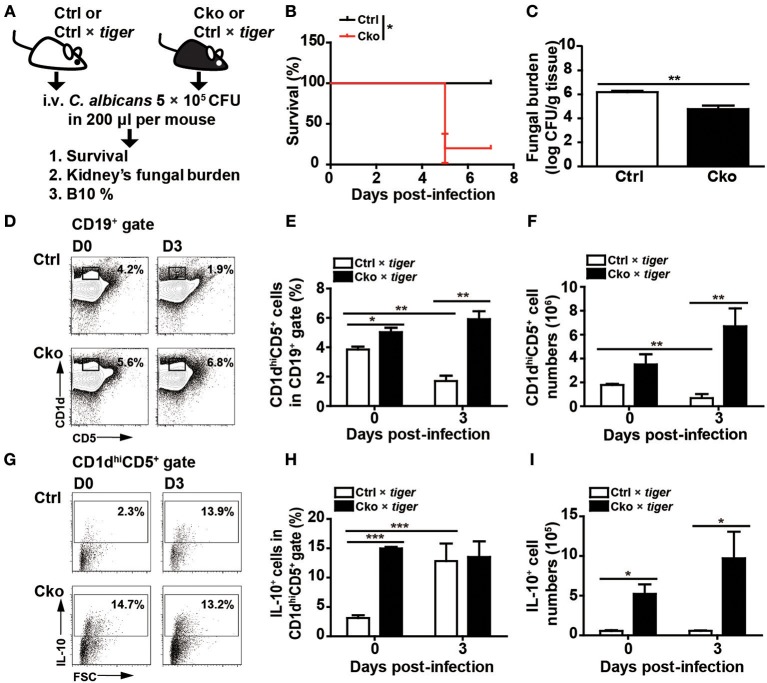
Blimp-1-deficient mice had increased mortality in systemic candidiasis. **(A)** Schematic design for the infection of Ctrl, Cko, Ctrl × *tiger*, and Cko × *tiger* mice with 5 × 10^5^ CFU of *C. albicans* by intravenous injection. **(B)** The survival rates of Ctrl and Cko mice were recorded (*n* = 5 mice per group). The differences in survival rates were analyzed by a log-rank (Mantel-Cox) test. **(C)** Three days after infection, the fungal burden in the kidneys of Ctrl and Cko mice was quantified, and the resulting data are shown as the log10 value (*n* = 4 mice per group). **(D–F)** The frequency **(D,E)** and numbers **(F)** of B10 cells in the Ctrl × *tiger* and Cko × *tiger* mice at day 3 post-infection. **(G–I)** The frequency **(G,H)** and numbers **(I)** of IL-10^+^ (GFP^+^) B10 cells in the Ctrl × *tiger* and Cko × *tiger* mice at day 3 post-infection. Splenocytes were stimulated with LPS for 5 h before cell surface staining (*n* = 3 mice per group in **D–I**). Results are from at least two independent experiments and were analyzed by using an unpaired Student's *t*-test. Data are the mean ± SEM. ^*^p < 0.05, ^**^p < 0.01, and ^***^p < 0.001.

Bregs have been implicated in modulating viral, parasitic, and bacterial infections (13–15). We sought to examine whether Bregs in the presence or absence of Blimp-1 affect fungal infections. We intravenously infected Ctrl and Cko mice (or Ctrl × *tiger* and Cko × *tiger* mice) with *C. albicans*, and recorded the resulting survival over time (Figure 4A). Our results show that Cko mice had increased mortality, as compared with Ctrl mice (Figure 4B). We also assessed the fungal burden, which is known to correlate with mortality in most cases (41), in the kidneys of Ctrl and Cko mice 3 days after infection. Unexpectedly, the Cko mice had a lower fungal burden, as compared with the Ctrl mice, despite having a higher mortality rate (Figure 4C). Similarly, milder gross pathology of the kidneys was found in the *C. albicans*-infected Cko mice as compared with infected Ctrl mice (Supplementary Figure 2). Moreover, after *C. albicans* infection, the frequency and number of splenic B10 cells were both lower in Ctrl × *tiger* mice, whereas the Cko × *tiger* mice still had relatively higher frequency and number of B10 cells compared with uninfected animals (Figures 4D–F).

Although there was a significant increase in the frequency of GFP^+^ IL-10-expressing B10 cells in the spleens of Ctrl × *tiger* mice after *C. albicans* infection (Figures 4G,H), the number of splenic IL-10-expressing B10 cells remained similar in Ctrl × *tiger* mice after infection (Figure 4I). However, the frequency of splenic GFP^+^ IL-10-expressing B10 cells in Cko × *tiger* mice was unchanged after infection, whereas the number of these cells was significantly higher than in Ctrl × *tiger* mice (Figures 4G–I). Thus, after *C. albicans* infection, Cko mice have more IL-10-expressing B10 cells in the spleen and a lower fungal burden in the kidneys but a more severe mortality rate.

**Figure 5:**
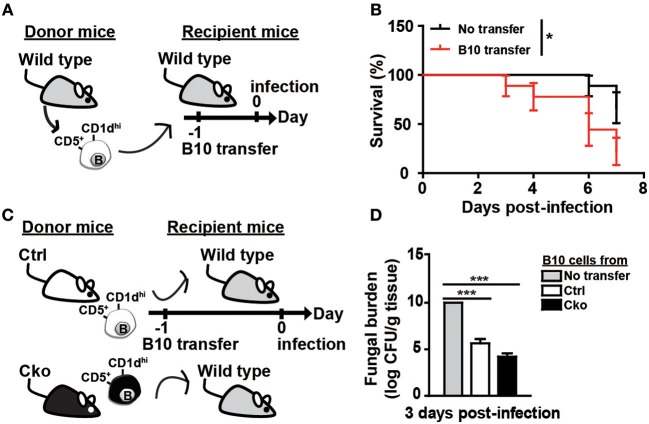
B10 cells promoted the clearance of *C. albicans*. **(A)** Adoptive transfer of B10 cells into wildtype mice. Splenic B10 cells were purified from wildtype mice by cell sorting. Recipient mice were given either PBS or 1 × 10^6^ B10 cells at 1 day before *C. albicans* infection. **(B)** The survival rates of the mice in **(A)** were recorded (*n* > 8 mice per group). The differences in survival were analyzed by a log-rank (Mantel-Cox) test. **(C)** Purified splenic B10 cells from Ctrl or Cko mice were adoptively transferred into wildtype recipient mice as described in **(A)**. **(D)** The fungal burden in the kidneys was quantified in day 3 post-infection (*n* = 3 mice per group). Results are from at least two independent experiments and were analyzed using an unpaired Student's *t*-test. Data are the mean ± SEM. ^*^p < 0.05 and ^***^p < 0.001.

To further clarify the roles of B10 cells in *C. albicans* infection, we adoptively transferred B10 cells from wildtype C57/BL6 mice into wildtype recipient C57/BL6 mice 1 day before infection (Figure 5A). Remarkably, the transfer of B10 cells significantly decreased the survival rate in the recipient mice (Figure 5B). A previous study showed that *Il-10* KO mice have a reduced kidney fungal burden, suggesting that IL-10 facilitates systemic candidiasis (42). To further understand the role of Blimp-1 in B10 cells in fungal infection, we adoptively transferred B10 cells from Ctrl or Cko mice into wildtype C57/BL6 mice. Fungal loads in the kidneys of recipient mice were assessed 3 days later (Figure 5C). Compared with recipient mice that did not receive B10 cells, there was a significantly reduced fungal burden in the recipient mice that received a transfer of Ctrl B10 cells 3 days after infection, whereas comparable fungal burden was found in the mice that received B10 cells transferred from Ctrl or Cko mice (Figure 5D). These data suggest that the function of B10 cells in fungal clearance may be independent of Blimp-1.

### B10 Cell Differentiation Following Stimulation Requires Blimp-1

**Figure 6:**
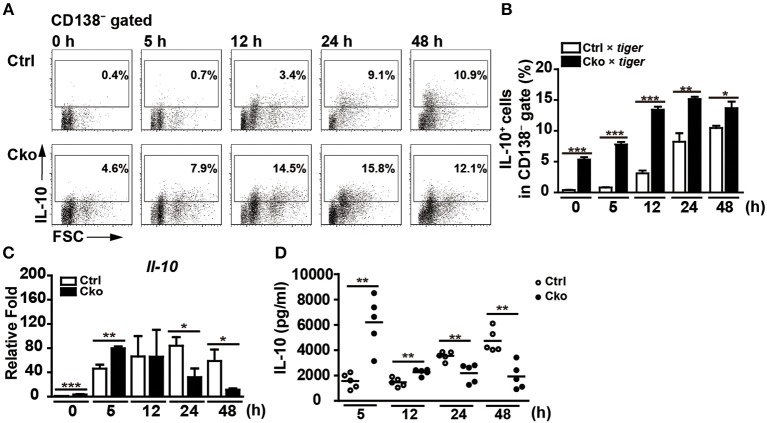
Blimp-1-deficient B10 cells stimulated in culture showed disturbed IL-10 production. **(A,B)** CD19^+^CD1d^hi^CD5^+^ B10 cells were sorted from Ctrl × *tiger* and Cko × *tiger* mice and then stimulated with LPS (10 μg/ml) for the indicated lengths of time. FACS analysis showing the frequency of IL-10^+^ (GFP^+^) cells in the CD138^−^ gate **(A)**. Statistical results from two independent biological repeats of the experiment described in **(A)** are shown **(B)**. **(C,D)** The *Il-10* mRNA levels in stimulated B10 cells **(C)** and IL-10 secretion by stimulated B10 cells **(D)** from the culture described in **(A)** were detected by RT-qPCR and ELISA, respectively (*n* = 3–8 mice per group). Data were analyzed using an unpaired Student's *t*-test and are the mean ± SEM. ^*^p < 0.05, ^**^p < 0.01, and ^***^p < 0.001.

B10 cells are able to differentiate into antibody-producing cells upon stimulation with LPS (35). Interestingly, a subset of plasmablasts, called regulatory plasmablasts, has been reported to express both Blimp-1 and IL-10 during *Salmonella* infection and in EAE (1, 6). Thus, we next examined whether a lack of Blimp-1 in B10 cells affects the generation of IL-10-producing regulatory plasmablasts. Sorted CD19^+^CD1d^hi^CD5^+^ B10 cells from Ctrl × *tiger* or Cko × *tiger* mice were stimulated with LPS. A low frequency of B10 cells from the Ctrl × *tiger* mice expressed detectable levels of GFP (IL-10) before stimulation (Figures 6A,B), but the frequency of GFP^+^ (IL-10^+^) cells in the CD138^−^ gate increased in a time-dependent manner following stimulation (Figures 6A,B). Consistent with published previously results (29, 35), we found that a fraction of sorted B10 cells differentiated into CD138^+^ plasmablasts after LPS stimulation (Supplementary Figure 3A). At 48 h after stimulation, 23.6% of the GFP^+^ (IL-10^+^) B10 cells from Ctrl × *tiger* mice were CD138^+^ (Supplementary Figure 3B). Consistently, the frequency of GFP^+^ (IL-10^+^) B cells was significantly higher in unstimulated B10 cells from Cko × *tiger* mice, as compared with that in similarly treated B10 cells from Ctrl × *tiger* mice (Figures 6A,B). Furthermore, we again found that a higher frequency of Blimp-1-deficient B10 cells were GFP^+^ (IL-10^+^) at all timepoints (Figures 6A,B), but Blimp-1-deficient B10 cells failed to differentiate into CD138^+^ regulatory plasmablasts after LPS stimulation (Supplementary Figures 3A,B). These results suggest that Blimp-1 in B10 cells is also required for the differentiation of B10 cells into IL-10-producing regulatory plasmablasts.

Although populations of B10 cells lacking Blimp-1 have more IL-10-producing cells, they fail to differentiate into IL-10-producing regulatory plasmablasts. We next examined the effect of Blimp-1 on IL-10 production following the stimulation of isolated B10 cells in culture. As shown in Figure 6C, the *Il-10* mRNA levels were higher at 0 and 5 h after stimulation with LPS in Cko B10 cultures, but after 12 h, the *Il-10* mRNA levels in these cultures were significantly reduced as compared with those in Ctrl B10 cultures. Likewise, higher IL-10 levels are present in the supernatant of LPS-stimulated Cko B10 cultures at 5 and 12 h post-stimulation, but LPS-stimulated Cko B10 cells produced less IL-10 at 24 and 48 h post-stimulation (Figure 6D) compared with LPS-stimulated Ctrl B10 cells. As a portion of plasmablasts also expressed substantial amounts of IL-10, our data suggest that Blimp-1-deficient B10 cells may eventually lose their ability to produce IL-10 once they are stimulated for differentiation.

### Blimp-1 Aids STAT3 in Regulating *Il-10* Transcription

**Figure 7:**
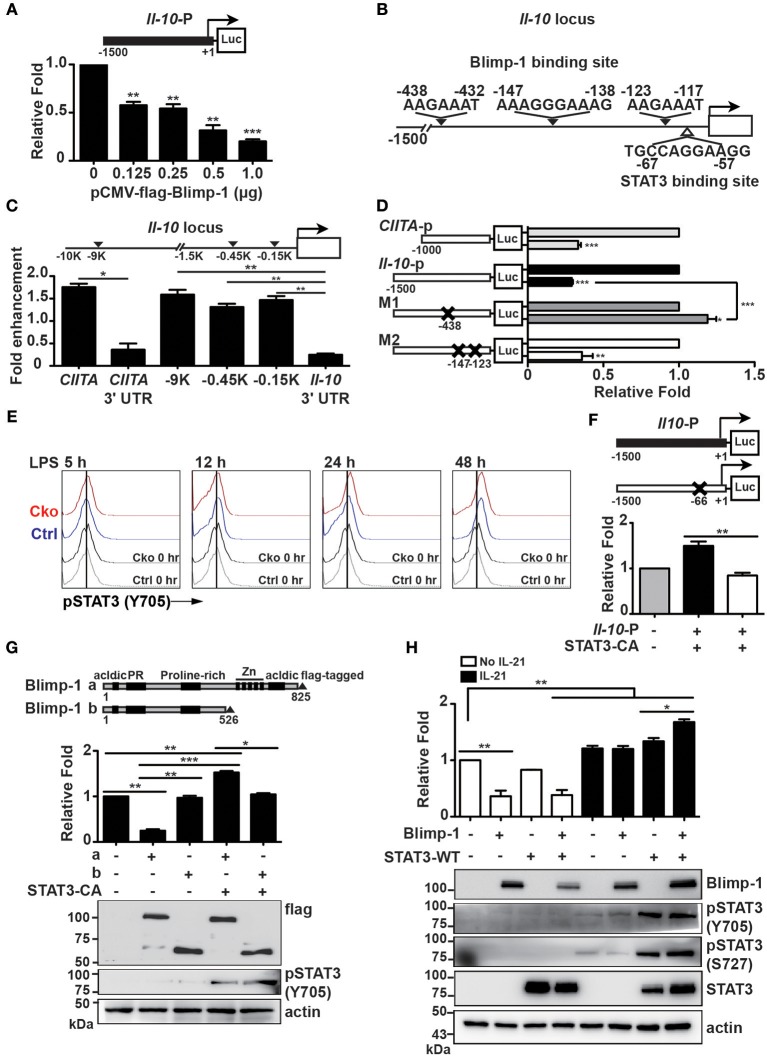
Blimp-1 collaborated with STAT3 to upregulate *Il-10* expression. **(A)** Relative fold differences of the *Il-10* promoter-dependent luciferase reporter activities repressed by the indicated doses of Blimp-1 expression plasmid in Raji B cells at 48 h post-transfection. **(B)** Schematic representation of predicted Blimp-1- and STAT3-binding sites on the *Il-10* promoter. **(C)** ChIP analysis of Blimp-1 binding to the *Il-10* promoter in LPS-stimulated splenic B cells. The binding of Blimp-1 to −9 kb of TSS and *CIITA* promoter III was used as the positive control, while the CIITA 3'UTR and *Il-10* 3'UTR served as negative controls. **(D)** *Il-10* promoter-dependent luciferase reporters carrying mutations in −438–432 bp of TSS (M1) or in −147 to −138 bp and −123 to −117 bp of TSS (M2) were used in co-transfection in luciferase reporter assays. The *CIITA* promoter III was used as the positive control. **(E)** STAT3 was phosphorylated at tyrosine 705 in splenic B10 cells purified from Ctrl or Cko mice and stimulated with LPS at the indicated timepoints. **(F)** Raji B cells were co-transfected with wildtype or STAT3-binding-site-mutated *Il-10* promoter-dependent luciferase constructs along with an expression plasmid encoding a constitutively active form of STAT3 (STAT3-CA). **(G)** Plasmids encoding STAT3-CA and full-length (a) or a truncated form of Blimp-1 lacking the DNA binding domain (b) were used in the *Il-10* promoter-dependent luciferase reporter assay. The expression of Blimp-1 and phosphorylated STAT3 (Y705) was confirmed by immunoblotting. **(H)** Luciferase reporter assays were conducted in Raji B cells co-transfected with the *Il-10* promoter-dependent luciferase reporter and the indicated plasmids in the presence or absence of IL-21 (20 μg/ml) treatment for 48 h. The expression of Blimp-1 and pSTAT3 (Y705 and S727) was confirmed by immunoblotting. Results are from three independent experiments and were analyzed by using an unpaired Student's *t*-test. Data are the mean ± SEM. ^*^p < 0.05, ^**^p < 0.01, and ^***^p < 0.001.

In Th1 cells, Blimp-1 binds to −9 kb of the transcriptional start site (TSS) of the *Il-10* gene locus to regulate *Il-10* transcription (43). It has also been reported that Blimp-1 directly binds to the *Il-10* locus at intron 1 with IRF4 to regulate *Il-10* expression in Tregs (23). Because the expression of IL-10 is increased in *Prdm1*-deficient B10 cells, we wondered whether Blimp-1 represses *Il-10* transcription. A luciferase reporter fused with −1.5 kb of the *Il-10* gene was generated and used for luciferase reporter assays via its co-transfection with a Blimp-1 expression plasmid into Raji B cells. We found that Blimp-1 represses *Il-10* promoter driven-luciferase activity in a dose-dependent manner (Figure 7A). Based on known conserved Blimp-1-binding sites (20, 44, 45), we found three putative Blimp-1-binding sites in the *Il-10* promoter region (Figure 7B). By performing ChIP analyses using chromatin from LPS-stimulated splenic B cells and anti-Blimp-1 antibody, we found that Blimp-1 directly binds to these putative sites at −0.45 and −0.15 kb of the TSS (Figure 7C). The binding of Blimp-1 to −9 kb of the *Il-10* TSS and to *CIITA* promoter III (43, 46) was used as the positive control, and *CIITA* 3′UTR and *Il-10* 3′UTR served as the negative control (Figure 7C). To determine whether these Blimp-1-binding sites are required for the regulation of *Il-10* transcription, we generated *Il-10* promoter-driven luciferase constructs carrying disrupted Blimp-1-binding sites: M1 with mutated −438 to −432 bp of the TSS, and M2 with mutated −147 to −138 bp and −123 to −117 bp of the TSS (Figure 7D). Results from our luciferase reporter assays demonstrate that M1 displayed an abolished Blimp-1-mediated repression of *Il-10* transcription (Figure 7D), implying that −0.45 kb of TSS is the major Blimp-1 suppressive site on the *Il-10* promoter.

Given that *Il-10* mRNA was present in a subset of IL-10-producing plasmablasts in which Blimp-1 is expressed, we hypothesized that Blimp-1 may work together with another transcription factor to positively regulate *Il-10* transcription after B10 cells are stimulated. Indeed, in T cells, Blimp-1 can work together with c-Maf or IRF4 to positively regulate IL-10 production (23, 43). Previous studies have demonstrated that STAT3 activation is required for LPS-induced IL-10 expression in B cells (47, 48). As such, we speculated that Blimp-1 may cooperate with the activated STAT3 to upregulate *Il-10* transcription. To investigate this possibility, the levels of STAT3 phosphorylation at tyrosine 705 site (Y705), which represents the activated form of STAT3 (49), in Ctrl and Cko B10 cells following LPS stimulation were examined by FACS analysis. We found that STAT3 was activated at similar levels in Ctrl and Cko B10 cells starting from 12 h after LPS stimulation (Figure 7E). A putative STAT3-binding site was found in the *Il-10* promoter at −67 to −57 bp of the TSS (Figure 7B). To determine whether *Il-10* expression can be affected by STAT3, we co-transfected Raji B cells with a plasmid encoding a constitutively active form of STAT3 (STAT3-CA) and an *Il-10* promoter-driven luciferase reporter construct or a luciferase reporter carrying a mutated STAT3-binding site. As shown in Figure 7F, the overexpression of STAT3-CA induced luciferase activity driven by the *Il-10* promoter, except when the putative STAT3-binding site was mutated.

To determine if there is any crosstalk between Blimp-1 and STAT3 in the transcription of *Il-10*, we co-transfected *Il-10-*luciferase reporter with the STAT3-CA expression vector and constructs encoding either full-length Blimp-1 or Blimp-1 lacking the Zn finger DNA-binding domain at its C-terminus. We found that Blimp-1 lacking the DNA-binding domain was not able to suppress *Il-10* promoter-driven luciferase activity (Figure 7G). Further, the combination of STAT3-CA and full-length Blimp-1 stimulated *Il-10* promoter-driven luciferase activity, but STAT3-CA was not able to effectively activate *Il-10* promoter-driven luciferase activity when the DNA-binding domain of Blimp-1 was not present (Figure 7G), suggesting that STAT3 requires Blimp-1 binding to DNA to activate *Il-10* transcription.

Previous studies demonstrated roles for IL-21 in promoting STAT3 phosphorylation in both human and murine B cells and in inducing IL-10 production in B10 cells (9, 50–52). We examined whether IL-21 could induce *Il-10* promoter-driven luciferase activity and whether it involves the collaboration of Blimp-1 and STAT3. We found that IL-21 induced both *Il-10* promoter-driven luciferase activity and STAT3 phosphorylation (Figure 7H). Notably, IL-21 induced higher levels of *Il-10* promoter-driven luciferase activity in the presence of both Blimp-1 and STAT3 (Figure 7H). Together, these data suggest dual roles for Blimp-1 in the regulation of *Il-10* transcription in B10 and activated B10 cells; that is, Blimp-1 directly represses *Il-10* in normal B10 cells but, after B10 cell stimulation, the newly phosphorylated STAT3 cooperates with Blimp-1 to upregulate *Il-10* transcription.

## Discussion

Traditionally, B cells were thought to play a positive role in controlling immune responses by producing antibody and serving as antigen-presenting cells. Recently, evidence has demonstrated an additional immunosuppressive role for B cells in maintaining immune tolerance to control inflammatory and autoimmunity diseases, such as EAE, CIA, and colitis (7, 38, 53, 54). In particular, CD1d^hi^CD5^+^ B10 cells are functionally characterized by their ability to produce IL-10 to provide immune suppression in inflammatory and autoimmunity diseases (5, 38, 55). The mechanisms controlling B10 cell generation and subsequent IL-10 production are largely unknown. Our results here revealed a dual role for Blimp-1 in IL-10 expression in B10 cells and plasmablasts. In agreement with a previous study (35), we found that *Prdm1* expression is higher in IL-10^+^ B cells as compared with IL-10^−^ B cells after LPS or anti-CD40 antibody stimulation. Additionally, we observed a time-dependent induction of Blimp-1^+^ B10 cells after LPS stimulation. *Prdm1* and *Irf4* expression have been reported to induce IL-10 production (23). In the present study, a portion of splenic B10 cells that had differentiated into CD138^+^ plasmablasts in culture after LPS stimulation had the capacity to produce IL-10. Notably, IL-10-producing plasmablasts expressing both *Prdm1* and *Irf4* have been reported before in the draining lymph nodes of an animal model of EAE progression (6). However, in that report, only *Irf4* was critical for IL-10 production by plasmablasts. These discrepancies between our findings may reflect the different sources of B cells and stimuli used in culture. Nevertheless, given that B10 cells can be induced by different signal pathways based on the inflammatory environment, it is conceivable that various factors may participate in the development and function of B10 cells.

Blimp-1 is well-known as a transcriptional repressor in controlling cell differentiation. Our data revealed a unique function of Blimp-1 in collaborating with activated STAT3 to upregulate *Il-10* in B cells. Additional work is needed to determine the mode of collaborative action of Blimp-1 and STAT3 for *Il-10* upregulation in stimulated Bregs. In Th2 cells, the IL-10-induced activation of STAT3 induced *Prdm1* expression (56). Notably, we found that STAT3 was not activated in B10 cells before stimulation. It is thus likely that this IL-10/STAT3/Blimp-1 axis may exist as a positive regulatory loop to amplify the production of IL-10 in stimulated B10 cells or plasmablasts. In T cell subsets, Blimp-1 has been shown to regulate Treg homeostasis but is dispensable for Treg function in the suppression of colitis and the generation of Tregs (22, 23). Additionally, *Irf4* was found to be required for the generation of Blimp-1^+^ Tregs and to collaborate with Blimp-1 to regulate the Treg gene expression program (23). In B cells, *Irf4* upregulates *Prdm1* to promote the transition of germinal center B cells to plasma cells (57). Moreover, *Irf4* has been shown to be expressed in B10 cells (35). Together, these findings support a further examination into the regulatory relationship of *Irf4*, Blimp-1, and activated STAT3 in the transcription of *Il-10* in Bregs and plasmablasts.

We found that B10 cells from Cko mice failed to suppress the proliferation of anti-CD3- and anti-CD28-stimulated naïve CD4^+^ T cells. In addition, Ctrl × *Il-10* KO B10 cells were still able to effectively suppress the proliferation of anti-CD3- and anti-CD28-primed naïve CD4^+^ T cells. It is well-established that IL-10 produced by Bregs dampens the differentiation of CD4^+^ T cells into Th1 and Th17 subtypes and induces Tregs (3, 58). The anti-inflammatory cytokines IL-35 and TGF-β produced by Bregs are also associated with Breg functions, such as the inhibition of Th1 and Th17 responses (1, 8). Our results suggest that IL-10 produced by B10 cells is dispensable for the suppression of T cell proliferation and that Blimp-1 in B10 cells contributes to this suppression function independently of IL-10. The Blimp-1-dependent mediators controlling the suppression of T cell proliferation should be investigated in future studies. Nevertheless, our microarray analysis showed that genes involved in “T cell selection” and “leukocyte migration,” including *Bcl2, Fas, Il-6*, and *Spn*, are differentially expressed in stimulated Cko B10 cells. It requires further study to determine whether those identified genes are involved in the suppression of T cell proliferation. Further, it has been shown that IL-21-induced human Bregs express granzyme B that contributes to the suppression of T-cell proliferation (59). Additionally, a subset of human Bregs that express CD39 and CD73 are able to generate adenosine 5′-monophosphate (5′-AMP) and adenosine that execute the suppression function of T cell activation (60).

Although IL-10 is an important immunomodulator in viral, bacterial, and parasitic infections, only a few studies have investigated the IL-10 released by Bregs in the control of infectious diseases. Regarding viral infection, IL-10-producing B cells were reported to inhibit hepatitis B virus (HBV)-specific CD8^+^ T cell responses, and the frequency of IL-10- producing B cells was found to be correlated with HBV pathogenesis (14). In *Listeria* infection, previous work indicated that B10 cells inhibit CD4^+^ T cell expansion and bacterial clearance by macrophages through dampening the production of IFN-γ and TNF-α (15). These results demonstrate a pathogenic role for Bregs in infectious diseases, in contrast to the more commonly reported anti-inflammatory and -autoimmune effects of Bregs. The mechanisms causing the deleterious effects of Bregs during infectious diseases are largely elusive. These functions could be dependent on the cognate interactions between CD4^+^ T cells and B10 cells because B10 cells that are deficient in IL-10 or IL-21 receptor expression did not affect *Listeria* clearance (15).

Regarding fungal infection, IL-10 has been reported to inhibit the defense against *C. albicans* infection by reducing the activation of IFN-γ producing Thl cells and macrophages, leading to impaired fungal clearance (61). Additionally, mice lacking *Il-10* are resistant to acute systemic candidiasis, as manifested by a lower fungal burden in kidneys (42). However, neither of these studies investigated the potential involvement of IL-10-producing B cells. Here, we found that the frequency and number of B10 cells are both significantly decreased in Ctrl mice after *C. albicans* infection, in contrast with the observed expansion of B10 cells following *Listeria* infection (15). However, the number of splenic B10 cells and IL10^+^ B10 cells were both significantly increased in Cko mice after *C. albicans* infection, which is linked with the higher mortality and lower fungal burden in infected Cko mice. Furthermore, *C. albicans*-infected mice that received a transfer of B10 cells also showed an increased mortality and reduced fungal burden as compared with the PBS transfer group. Together, these results suggest that, regardless of their positive role in the clearance of fungal load, B10 cells promote mortality due to fungal infection. Surprisingly, mice that received a transfer of Blimp-1-deficient B10 cells had a fungal load comparable with that of Ctrl B10 cell-recipient mice, despite Cko B10 cells expressing altered levels of IL-10. We propose that this may have partly resulted from an impaired differentiation of plasmablasts/plasma cells from Cko B10 cells. The role of antibody-mediated immunity in host defense against fungal infections is still controversial, especially in systemic *C. albicans* infection (62, 63). Cko mice were not able to generate detectable levels of antigen-specific antibody. Nevertheless, adoptive transfer of serum from *C. albicans* infected wildtype C57BL/6 mice was able to significantly improve the survival rates of *C. albicans* infected Cko mice (Supplementary Figures 4A,B). Therefore, our data suggest that a lack of antibody as well as increased generation of B10 cells may be pathogenic for fungal infection in Cko mice. Our data also suggested that the enhanced generation of B10 cells in Cko mice or the extra dose of B10 cells resulting from the B10 transfer experiment may cause a “cytokine storm” or immunopathology that is harmful to the hosts. Targeting Bregs as a therapeutic strategy has been proposed; however, our results suggest that further caution is required when utilizing the adoptive transfer of Bregs for the treatment of certain diseases.

In conclusion, our results demonstrate a complex role for Blimp-1 in the regulation of IL-10 expression in various stages of Bregs. In the steady state, Blimp-1 directly represses *Il-10* in B10 cells, but, after stimulation, Blimp-1 cooperates with phosphorylated STAT3 to activate *Il-10* transcription. Thus, Blimp-1 restrains the generation of B10 cells as well as the IL-10 production in B10 cells, but it promotes the differentiation of B10 cells into IL-10-secreting plasmablasts. Our data are also the first to show that B10 cells may have a pathological role in fungal infections despite improving the clearance of fungi in a Blimp-1-independent manner. Thus, Blimp-1 has a dynamic role in Breg generation and function.

## Data Availability

The raw data supporting the conclusions of this manuscript will be made available by the authors, without undue reservation, to any qualified researcher.

## Ethics Statement

All mice were housed in a specific pathogen-free facility in the Institute of Cellular and Organismic Biology at Academia Sinica and handled in accordance with the guidelines of the Institutional Animal Care and Use Committee.

## Author Contributions

Y-HW and K-IL conceived and designed the study. Y-HW, D-YT, Y-AK, T-TY, and K-HH performed the experiments. Y-HW, D-YT, Y-AK, I-YL, and K-IL analyzed the data. Y-HW and K-IL wrote the manuscript.

### Conflict of Interest Statement

The authors declare that the research was conducted in the absence of any commercial or financial relationships that could be construed as a potential conflict of interest.
