# Supplementary material for: Blimp-1 Contributes to the Development and Function of Regulatory B Cells
Source: Front Immunol. 2019 Aug 14;10:1909. doi: 10.3389/fimmu.2019.01909 (PMC6702260; doi:10.3389/fimmu.2019.01909)
Supplement: Supplementary file 2 [file Presentation_1.pdf]

Supplementary Material

Blimp-1 Contributes to the Development and Function of Regulatory B Cells

Ying-Hsiu Wang[1,2], Dong-Yan Tsai[2], Yi-An Ko[2], Tsan-Tzu Yang[2,3], I-Ying Lin[2], Kuo-Hsuan Hung[2], and Kuo-I Lin[1, 2, 3, *]

[1]Graduate Institute of Life Sciences, National Defense Medical Center, Taipei 114, Taiwan;

[2]Genomics Research Center, Academia Sinica, Taipei 115, Taiwan;

[3] Graduate Institute of Immunology, College of Medicine, National Taiwan University, Taipei 110, Taiwan

***Corresponding author:**

Kuo-I Lin (ORCID 0000-0003-4477-0798)

Genomics Research Center, Academia Sinica, 128 Academia Road, Sec. 2, Nankang Dist., Taipei 115, Taiwan

Phone: +886-2-2787-1253

Fax: +886-2-2789-9931

Email: kuoilin@gate.sinica.edu.tw

SUPPLEMENTARY TABLE AND FIGURES

SUPPLEMENTARY TABLE 1. Differentially expressed genes in IL-10^{+} and IL-10^{-} splenic B cells stimulated with LPS or anti-CD40 antibody.

SUPPLEMENTARY TABLE 2. Microarray analysis of the expression of genes with more than 1.5-fold changes in Cko B10 cells before and 48 h after anti-CD40 stimulation.

SUPPLEMENTARY FIGURE 1. Proliferation and apoptosis rates in Ctrl and Cko B10

cells after stimulation.

SUPPLEMENTARY FIGURE 2. Gross pathology of kidneys from Ctrl and Cko mice infected with *C. albicans*.

SUPPLEMENTARY FIGURE 3. B10 cells differentiated into $CD138^{+}$ plasmablasts after LPS stimulation.

SUPPLEMENTARY FIGURE 4. Adoptive transfer of serum from *C. albicans* infected mice reduced the mortality caused by *C. albicans* infection in Cko mice.

SUPPLEMENTARY TABLE 1. Differentially expressed genes in IL-10⁺ and IL-10⁻ splenic B cells stimulated with LPS or anti-CD40 antibody. Microarray analysis revealed the up- and down-regulated genes in IL-10⁺ splenic B cells after stimulation, as compared with IL-10⁻ splenic B cells. Thirty-four and four genes were up- and down-regulated, respectively (fold changes of ≥ 1.8), in IL-10⁺ B cells as compared with IL-10⁻ B cells. Two independent experiments were performed.

	Experiment 1 LPS		Experiment 1 anti-CD40 antibody		Experiment 2 LPS		Experiment 2 anti-CD40 antibody				
Probe Set ID	Fold Change	Regulation	Fold Change	Regulation	Fold Change	Regulation	Fold Change	Regulation	Gene Symbol	Entrez Gene	Alignments
1450330_at	5.29521	up	12.714221	up	4.292656	up	10.32317	up	*Il10*	16153	chr1:131019846-131024970 (+) // 99.09 // qE4
1422707_at	4.3830457	up	2.0213315	up	3.1081445	up	3.0763001	up	*Pik3cg*	30955	chr12:32173132-32208470 (-) // 88.11 // qA3
1439897_at	4.304222	up	5.0168533	up	3.3988318	up	3.5492852	up	*Nebl*	74103	chr2:17375018-17390982 (-) // 99.87 // qA3
1420425_at	4.285112	up	11.076121	up	3.0637705	up	6.451456	up	*Prdm1*	12142	chr10:44437174-44458748 (-) // 94.56 // qB2
1417995_at	3.292895	up	3.5207381	up	2.2172718	up	2.0241675	up	*Ptpn22*	19260	chr3:103860291-103912247 (+) // 94.29 // qF2.2
1417392_a_at	3.2892392	up	4.4193306	up	2.0447004	up	2.2043543	up	*Slc7a7*	20540	chr14:54369373-54409051 (-) // 90.25 // qC2
1429775_a_at	2.9582744	up	2.026032	up	1.8286815	up	4.024682	up	*Gpr137b*	83924	chr13:12616893-12650380 (-) // 99.48 // qA1
1428942_at	2.9540799	up	8.978006	up	2.6136837	up	6.330001	up	*Mt2*	17750	chr8:94172781-94173564 (+) // 93.29 // qC5
1436649_at	2.94711	up	2.548358	up	2.0841537	up	2.1637824	up	*Ikzf3*	22780	chr11:98464895-98466043 (-) // 84.31 // qD
1434252_at	2.945844	up	5.3405695	up	2.3646157	up	2.11213	up	*Tmcc3*	319880	chr10:94588022-94590098 (+) // 89.71 // qC2
1455660_at	2.8591983	up	4.974766	up	2.3839257	up	4.3152986	up	*Csf2rb*	12983	chr15:78350198-78350997 (+) // 97.78 // qE1
1422557_s_at	2.7599635	up	5.329776	up	2.2777429	up	3.9664156	up	*Mt1*	17748	chr8:94179141-94180327 (+) // 55.44 // qC5
1450297_at	2.7019446	up	2.4143407	up	3.1647139	up	1.9088011	up	*Il6*	16193	chr5:30013143-30019968 (+) // 90.85 // qB1

	Experiment 1 LPS		Experiment 1 anti-CD40 antibody		Experiment 2 LPS		Experiment 2 anti-CD40 antibody				
Probe Set ID	Fold Change	Regulation	Fold Change	Regulation	Fold Change	Regulation	Fold Change	Regulation	Gene Symbol	Entrez Gene	Alignments
1427577_x_at	2.6779115	up	2.2451954	up	2.065782	up	2.1219835	up	*Igk-V21*	16071	chr6:70371872-70726738 (+) // 50.13 // qC1
1450140_a_at	2.674659	up	5.1833787	up	2.5587728	up	3.4741762	up	*Cdkn2a*	12578	chr4:89274544-89294599 (-) // 89.22 // qC4
1416808_at	2.6027803	up	3.7149382	up	3.221675	up	2.3177993	up	*Nid1*	18073	chr13:13437601-13512275 (+) // 92.63 // qA1
1416066_at	2.5760148	up	2.4849408	up	1.9680418	up	2.3939195	up	*Cd9*	12527	chr6:125460265-125494765 (-) // 92.74 // qF3
1449152_at	2.5496545	up	5.7169304	up	2.9264941	up	2.2581322	up	*Cdkn2b*	12579	chr4:89306286-89310993 (-) // 99.2 // qC4
1420450_at	2.5078306	up	2.4896743	up	2.8194594	up	1.8753654	up	*Mmp10*	17384	chr9:7502341-7510238 (+) // 98.52 // qA1
1419298_at	2.4851675	up	3.0290012	up	2.3365417	up	3.9852161	up	*Pon3*	269823	chr6:5221350-5256278 (-) // 98.08 // qA1
1451951_at	2.362497	up	4.441163	up	2.8539584	up	1.9155523	up	*Igk*	243469	chr6:68256581-68256866 (+) // 38.61 // qC1
1419249_at	2.345807	up	2.1448216	up	2.2939608	up	5.3039994	up	*Cdk14*	18647	chr5:4803384-5380251 (-) // 97.47 // qA1
1424927_at	2.2323787	up	2.299978	up	2.0620165	up	1.8619931	up	*Glipr1*	73690	chr10:111985454-111997264 (-) // 91.1 // qD2
1427837_at	2.20593	up	4.8589444	up	2.3319187	up	3.8068845	up	*Igkv15-103*	692169	chr6:68437642-68437928 (+) // 52.31 // qC1
1427860_at	2.1379027	up	2.278748	up	1.9711353	up	2.2426953	up	*Igkv6-15*	108022	chr6:70406465-70406736 (-) // 86.73 // qC1
1420679_a_at	2.0804083	up	1.8736295	up	2.2257009	up	2.1644626	up	*Aig1*	66253	chr10:13652450-13868957 (-) // 96.23 // qA2
1455854_a_at	2.0626478	up	2.6412814	up	1.9874107	up	2.2677712	up	*Ssh1*	231637	chr5:113939764-113941100 (-) // 71.58 // qF
1423311_s_at	1.985859	up	3.9583142	up	2.306383	up	2.8652906	up	*Tpbg*	21983	chr9:85842379-85847055 (+) // 93.8 // qE3.1
1430523_s_at	1.9613429	up	2.916712	up	1.8423766	up	3.700013	up	*Iglv1*	16142	chr16:19084852-19085498 (-) // 99.82 // qA3
1416431_at	1.936019	up	2.074861	up	1.9593635	up	1.8447341	up	*Tubb6*	67951	chr18:67390729-67402752 (+) // 94.69 // qE1
1419665_a_at	1.8736795	up	3.4004576	up	3.0027506	up	2.764955	up	*Nupr1*	56312	chr7:126623247-126625475 (-) // 93.05 // qF3

	Experiment 1 LPS		Experiment 1 anti-CD40 antibody		Experiment 2 LPS		Experiment 2 anti-CD40 antibody				
Probe Set ID	Fold Change	Regulation	Fold Change	Regulation	Fold Change	Regulation	Fold Change	Regulation	Gene Symbol	Entrez Gene	Alignments
1447918_x_at	1.8658237	up	2.0910437	up	1.8439312	up	2.1277769	up	*Iglc2*	110786	chr16:19198400-19198554 (-) // 86.63 // qA3
1418340_at	1.8390578	up	2.1524723	up	3.4976294	up	9.314293	up	*Fcer1g*	14127	chr1:171229576-171234309 (-) // 88.6 // qH3
1424931_s_at	1.8093346	up	2.8896892	up	1.8624929	up	3.18194	up	*Iglc1*	16142	chr16:19061618-19085499 (-) // 99.77 // qA3
1455530_at	5.299976	down	3.403353	down	5.44376	down	2.872428	down	*Ighd*	380797	chr12:113406604-113416235 (-) // 71.12 // qF1
1422122_at	2.6667225	down	2.4160252	down	3.0335176	down	6.074062	down	*Fcer2a*	14128	chr8:3681805-3694174 (-) // 72.11 // qA1.1
1430558_at	2.506817	down	2.268199	down	2.5393598	down	1.9812241	down	*Zfp318*	57908	chr17:46413583-46414817 (+) // 100.0 // qC
1442544_at	2.0500302	down	2.530094	down	2.5675843	down	3.9537458	down	*Ighg1*	16017	chr12:113325242-113325950 (-) // 96.82 // qF1

SUPPLEMENTARY TABLE 2. Microarray analysis of the expression of genes with more than 1.5-fold changes in Cko B10 cells before and 48 h after anti-CD40 stimulation. B10 cells were sorted from Ctrl or Cko mice and subjected for anti-CD40 stimulation. Genes whose expression was changed for more than 1.5-fold in Cko B10 cells were listed in a separate Excel data sheet (Supplementary Table 2).

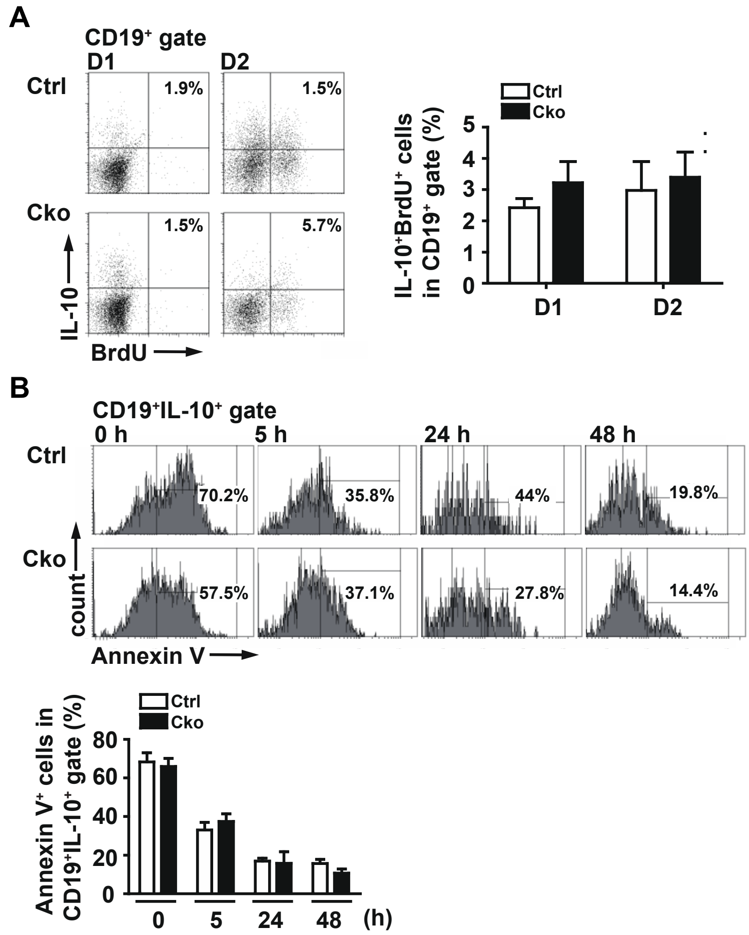

SUPPLEMENTARY FIGURE 1. Proliferation and apoptosis rates in Ctrl and Cko B10 cells after stimulation. (A) Splenic B cells from Ctrl or Cko mice were stimulated with LPS (10 µg/ml) for the indicated lengths of time as described previously. Cells were treated with 30 µM 5-bromo-2'-deoxyuridine (BrdU) for 2 h before being harvested. Data represent the $BrdU^+$ cells in the group of $CD19^+IL\text{-}10^+$ cells. (**B**) Cells were collected at the indicated timepoints and subjected to Annexin V staining. All antibodies and reagents are from BD Bioscience, and the staining procedure was performed in accordance with the manufacturer's instructions. Data are the mean ± SEM (n = 3−5 mice per group). At least two independent experiments were performed.

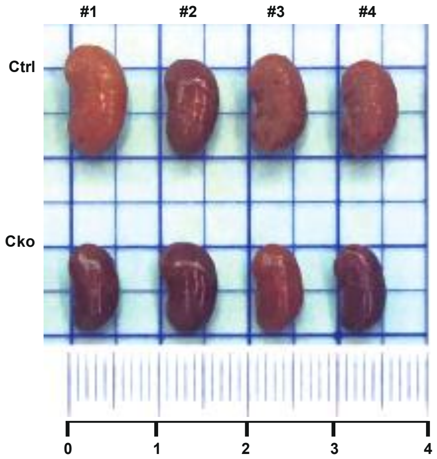

SUPPLEMENTARY FIGURE 2. Gross pathology of kidneys from Ctrl and Cko mice infected with *C. albicans*. Ctrl and Cko mice were infected with 5 × 10^5 CFU of *C. albicans* by intravenous injection. Three days after infection, kidneys from infected mice were removed for comparison (n = 4 mice per group).

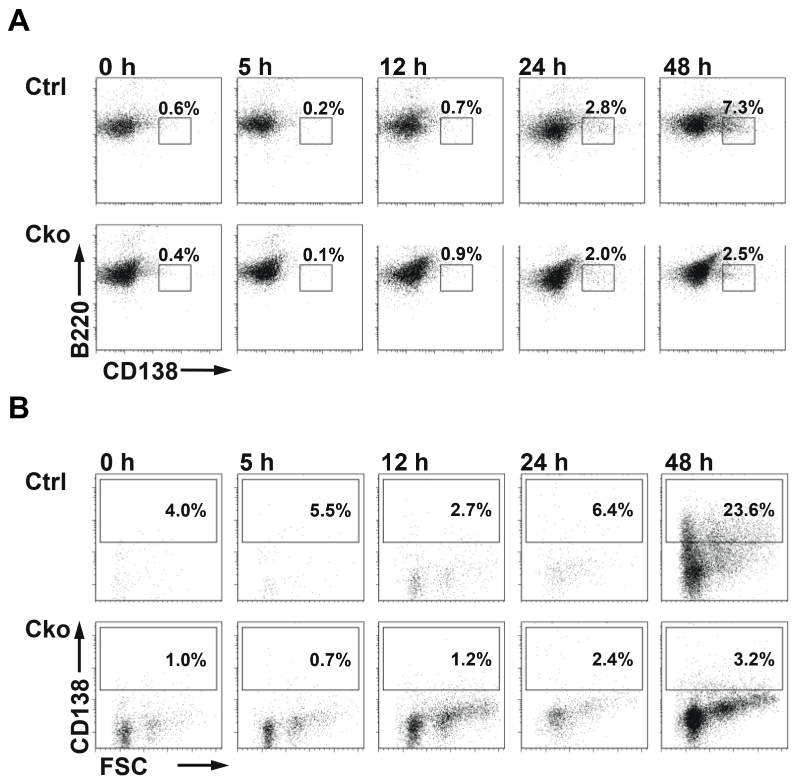

SUPPLEMENTARY FIGURE 3. B10 cells differentiated into CD138^{+} plasmablasts after LPS stimulation. CD19^{+}CD1d^{hi}CD5^{+} B10 cells were sorted from Ctrl × *tiger* or Cko × *tiger* mice and then stimulated with LPS (10 µg/ml) for the indicated lengths of time. **(A)** FACS analysis showing the frequency of CD138^{+} plasmablasts. **(B)** FACS showing the frequency of CD138^{+} in the IL-10^{+} gate. Data are the mean ± SEM (n = 4 mice per group). Two independent experiments were performed.

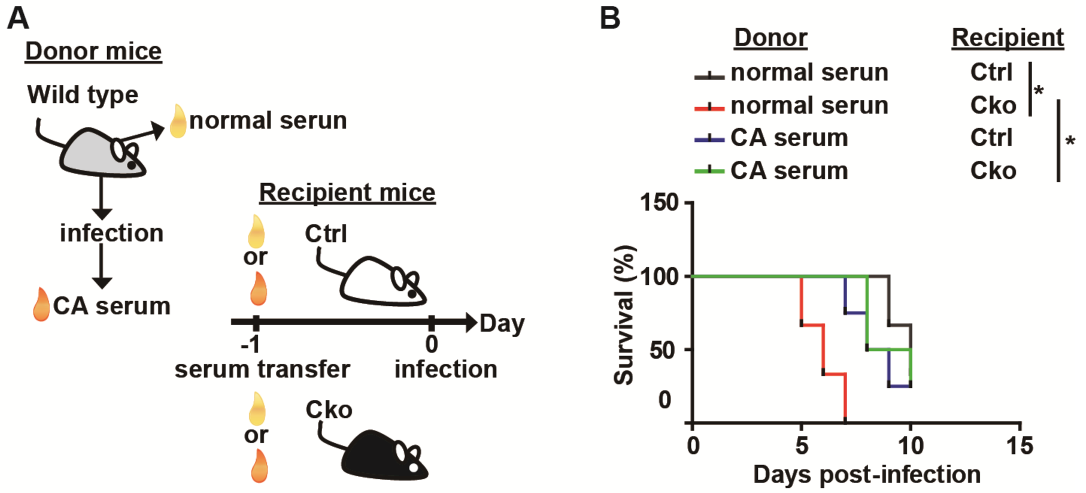

SUPPLEMENTARY FIGURE 4. Adoptive transfer of serum from *C. albicans* infected mice reduced the mortality caused by *C. albicans* infection in Cko mice. (A) Schematic design for transfer of serum collected from day 14 *C. albicans* infected (CA serum) or uninfected (normal serum) wildtype C57BL/6 mice into Ctrl or Cko mice, followed by challenge with 5×10^5 CFU of *C. albicans*.**(B)** The survival rate of Ctrl and Cko mice in **(A)** was recorded (n = 3−4 mice per group). The differences in survival rates were analyzed by a log-rank (Mantel-Cox) test. $^{*}p < 0.05$.
